# Formulating Treatment to Cure Alzheimer’s Dementia: Approach #2

**DOI:** 10.3390/ijms25063524

**Published:** 2024-03-20

**Authors:** Jeffrey Fessel

**Affiliations:** Department of Medicine, University of California, 2069 Filbert Street, San Francisco, CA 94123, USA; jeffreyfessel@gmail.com; Tel.: +415-563-0818

**Keywords:** Alzheimer’s dementia, treat to cure, treat operative causal elements, treat individual patients, individualized treatments, personalized medicine

## Abstract

There are two generic approaches to curing any medical condition. The first one treats every patient for all the known possible causes that contribute to pathogenesis; the second one individualizes potentially curative therapy by only identifying in each separate patient the components of pathogenesis that are actually operative and treating those. This article adopts the second approach for formulating a cure for Alzheimer’s dementia (AD). The components of AD’s pathogenesis are, in alphabetical order, as follows: circadian rhythm disturbances, depression, diabetes and insulin resistance, dyslipidemia, hypertension, inflammation, metabolic syndrome, mitochondrial dysfunction, nutritional deficiencies, TGF-β deficiency, underweight, vascular abnormalities, and Wnt/β-catenin deficiency. For each component, data are described that show the degree to which its prevalence is higher in patients with mild cognitive impairment (MCI) who did not revert to having normal cognition than in those who did because the former group is the pool of patients in which future AD may develop. Only addressing the components that are present in a particular individual potentially is a curative strategy. Published data indicate that curative therapy requires the number of such components that are addressed to be ≥3. Although structural brain changes cannot be directly addressed, the impaired neural tracts result from many of the reversible causal elements, so correcting them will benefit these tracts.

## 1. Introduction

Despite thousands of articles that detail its causal factors, there is still no cure for Alzheimer’s dementia (AD). A likely reason for this failure is that almost all clinical trials of potential treatments have applied single drugs. There are, however, two generic principles applicable to curing any medical condition. When applied to Alzheimer’s dementia (AD), the first of these principles would treat all the known possible causes that contribute to its pathogenesis. AD has 15+ causes (although the number of causes depends upon how they are counted); however, that number is high and potentially curative treatment would require an unmanageably high number of drugs. Also, in this first approach, a cure would apply to every patient; therefore, since causes of AD may differ from patient to patient, such a “one size fits all” approach may be inappropriate. The second generic principle identifies in each separate patient only the components of pathogenesis that are actually operative and treats those. Identifying these components can be difficult. However, for curing AD, identifying these components may be based upon the fact that many subjects (>30% in one large study [1]) with mild cognitive impairment (MCI) revert to having normal cognition (NC), while most do not. Addressing these causal elements, the prevalence of which is higher in patients who did not revert to NC than in those who did, is potentially a curative strategy because it is in the non-reverters that future AD may develop. Note that AD itself has never been reported to have spontaneous remission; were it to have occurred, the causal elements less prevalent in non-remitters than remitters would also point to potentially curative treatment of persistent AD. This article describes, as illustrated by Table 1, the second approach to curing AD and considers how to formulate curative therapy that is correct for individual patients, i.e., that which comprises personalized and individualized medicine.

There are two preliminary issues. First, any treatment for AD can only affect causal elements that are reversible; irreversible elements such as gender, prior education, and structural brain changes must be ignored. It will be contested that structural brain changes with impaired anatomy and physiology of the neural tracts may underlie dementia and must accordingly be the focus of curative treatment. It should be noted, however, that the impaired neural tracts result from many of the reversible causal elements, so correcting these will benefit the tracts. The second issue concerns the minimal number of conditions that need correcting in order to achieve a cure. That number must be established through clinical trials, but published data, which will be shown below, suggest it is ≥3.

In brief, the approach proposed here is based on the biological changes that occurred in an individual patient, which means that curing AD may require different treatments for different patients. The following sections show all the reversible, important causal elements that may require correction, and they are largely based upon data showing the causal elements of which the prevalence is higher in MCI patients who did not revert to NC than in those who did. Addressing a sufficient number of those elements in patients who have Alzheimer’s dementia should cure their dementia.

## 2. Depression and AD

Although studies show differences in the prevalence of depression in patients who progressed from having MCI to having AD, the general finding is that those individuals had an increased occurrence of baseline depression when contrasted with those having MCI who reverted to normal cognition. Among participants with mild cognitive impairment, 52.0% in midlife (<65 years) and 26.6% in later life (≥65 years) reverted to normal cognition after 4 years, and those whose age was <65 had more reversion to normal cognition after 4 years if they had had less depression at baseline [1]. Conversely, those with more depression at baseline subsequently over a 2 year period had more progression of MCI to dementia, with a hazard ratio (HR) of 4.8 [2]. A small study followed 105 persons with MCI and used the Montgomery–Asberg Depression Rating Scale (MADRS) [3]. At baseline, the 105 subjects had the MADRS score of 9.8; the 23 cases who had developed dementia after 3 years’ follow-up had the baseline MADRS score of 11.5 (*p* < 0.01). A similar observational study had 68 patients who progressed to dementia among the 279 patients with MCI; the odds ratio (OR) for baseline depression and subsequent dementia was 1.62 [4]. Mourao et al., in a meta-analysis of 18 studies that had 10,861 MCI subjects, found that in the group of MCI subjects progressing to dementia, those with depressive symptoms at baseline as compared to those without them had a pooled risk ratio (RR) of 1.28 (*p* = 0.003) [5]. In nine cohort studies of participants with MCI and depression, eight had an increased risk of progression to dementia, with a pooled RR of 1.35 [6].

There were discrepant findings in a 6-year follow-up of 441 community-dwelling persons with MCI, in which depression as measured by the Hamilton depression scale was no more common in those who progressed to dementia than in those with MCI who did not progress so [7]. That discrepant finding may relate to the population that was studied because a review of 14 published studies showed a 44.3% prevalence of depression in 1899 hospitalized patients with MCI but only 15.7% in 775 community-dwelling patients. There are many possible reasons for this difference; one reason may be that the reason for hospitalization was itself a risk factor for MCI, e.g., diabetes.

Data are mixed regarding whether treating depression, if present, contributes to the reversal of dementia. Persons who purchased antidepressants once (N = 687,552) had an increased rate of dementia compared to persons unexposed to antidepressants (N = 779,831). Nevertheless, the rate of dementia changed over time; therefore, during the initial prescription periods, the rate increased with the number of prescriptions, but continued treatment with antidepressants was associated with a reduction in the rate of dementia even if it was not to the same level as the rate for the general population [8]. This pattern was found for all classes of antidepressants (SSRIs, newer non-SSRI antidepressants, and older antidepressants). All findings were replicated in sub-analyses with Alzheimer’s disease as the outcome. Overall, the literature shows that while depression is associated with increased dementia, the use of antidepressants does not only protect against dementia but may even aggravate it. A population-based retrospective case–control analysis used the Taiwan National Health Insurance Research Database and identified two subsets as follows: 5394 cases, who had major depression and subsequently were diagnosed with dementia, and 5232 controls, who had major depression but no history of dementia [9]. The dementia patients were more likely to have diabetes, hypertension, stroke, and head injury, i.e., risk factors for dementia. In patients using tricyclic antidepressants, the OR for dementia was 0.24, but for those using selective serotonin reuptake inhibitors (SSRIs), the OR for subsequent dementia was 2.48. Another review of five studies involving 53,955 participants with major depression showed a far higher RR of 2.13 for dementia, which was 2.13 for tricyclic use and 1.75 for SSRIs [10]. A report of 18 studies involved 2,119,627 participants with a mean age ranging from 55 to 81 years; among those who had depression, antidepressant users also showed a significantly higher risk of dementia (RR = 1.37) and also of MCI (RR = 1.20) than the non-users [11]. Notably, both patients with depression who used antidepressants (RR = 1.50) and those who did not use antidepressants (RR = 1.3) also had significantly more dementia than the general population. An interesting report from Denmark showed that persons who purchased antidepressants once (N = 687,552) had an increased rate of dementia compared to persons unexposed to antidepressants (N = 779,831), and although continued long-term treatment with antidepressants was associated with a reduction in the rate of dementia, that rate remained above the rate for the general population [8]. This pattern was found for all classes of antidepressants (SSRIs, newer non-SSRI antidepressants, and older antidepressants). All findings were replicated in sub-analyses with Alzheimer’s disease as the outcome. Thus far, it seems that depressed persons have an increased rate of dementia and that using antidepressants either does not affect that rate or increases it. A study from Australia that commenced with 4922 cognitively normal men aged 71–89 included 388 with a past history of depression and 294 with current depression, and over an 8.9 years’ follow-up, 903 developed dementia [12]. The subhazard ratios (SHRs) of dementia for men with past and current depression were 1.3 (95% confidence interval (CI) = 1.0, 1.6) and 1.5 (95% CI = 1.2, 2.0). The use of antidepressants did not decrease this risk. The subhazard rates of dementia associated with questionable, mild-to-moderate, and severe depressive symptoms were 1.2, 1.7, and 2.1, respectively. Over an almost 14-year duration of follow-up, the percentages of patients remaining free of dementia were consistently higher for those who had never been depressed than for those with past depression who, in turn, had a higher rate than those with current depression. Although the authors concluded that “depression is more likely to be a marker of incipient dementia than a truly modifiable risk factor”, their data show that the respective adjusted subhazard ratios for dementia were 0.2 if with no depressive symptoms but using antidepressants, 1.1 if with questionable depressive symptoms and not using antidepressants, 1.4 if with questionable depressive symptoms but using antidepressants, 1.6 if with mild-to-moderate depressive symptoms but not using antidepressants, 2.5 if with mild-to-moderate depressive symptoms and using antidepressants, 1.5 if with severe depressive symptoms but not using antidepressants, and 4.8 if with severe depressive symptoms and using antidepressants. The interaction between the severity of depressive symptoms and the use of antidepressants with dementia risk was statistically significant (*p* < 0.05); the above data clearly show that antidepressant use is markedly associated with the development of dementia. The mean subhazard ratio for dementia if using antidepressants (bolded numbers) was 2.9 versus 1.4 if not using antidepressants (italicized numbers).

Anticholinergics for depression, such as amitriptyline and paroxetine, have been linked to a higher risk of dementia even when they were used up to 20 years beforehand. Thus, Richardson et al. examined the use of defined doses of anticholinergic drugs that had been prescribed 4–20 years before a diagnosis of dementia. A total of 14,453 (35%) cases and 86,403 (30%) controls were prescribed at least one anticholinergic drug with definite anticholinergic activity during the exposure period [13]. The adjusted odds ratio for any definite, anticholinergic drug was 1.11. Dementia was associated with an increasing average anticholinergic drugs’ score for drugs prescribed 4–20 years before a diagnosis of dementia. The risk of dementia increased with higher exposure to antidepressant, urological, and antiparkinsonian drugs with a high anticholinergic score, and this was also the case even for exposure 15–20 years before a diagnosis. However, not all anticholinergic drugs were associated with dementia; no link was seen between increased dementia risk and other anticholinergic drugs, such as antihistamines and medications for abdominal cramps.

In brief, depression per se is a risk factor for dementia, but the treatment of depression may actually aggravate the risk of dementia.

## 3. Diabetes, Insulin Resistance, and AD

A study that followed 500 subjects with normal cognition at baseline showed that the OR for subsequent MCI in 160 of them was 5.28 times in diabetics compared with 340 whose cognition remained normal, and another one that compared patients with MCI at baseline who progressed to dementia with non-progressors found that progression in those with diabetes was 3-fold higher than in those without diabetes [14]. The pooled RR was 1.92 in two reviews that analyzed 11 reports of patients with diabetes and MCI that progressed to dementia [6,15]. In another review of published studies on diabetic patients with MCI, their reduced rate of cognitive decline was clearly shown as being due to the treatment of diabetes since the RR in four studies was 0.53 when individuals taking medications were compared with those not taking them [16]. It is notable that prediabetes, defined as HbA1c 5.7–6.4%, did not predict subsequent dementia for the 11,656 participants of the Atherosclerosis Risk in Communities (ARIC) study [17]. In that study, an earlier age of onset of diabetes had the strongest association with dementia as follows: for onset before 60 years, HR 2.92; for onset at 60–69 years, HR 1.73; and for onset at 70–79 years, HR 1.23. Perhaps these differences stem from a longer duration of diabetes when its onset is earlier.

Regarding the mechanism for the association between diabetes and AD, a small but relevant study showed that those with MCI who progressed to dementia had a reduced cerebral metabolic rate of glucose metabolism, which is because cerebral insulin resistance is heightened in diabetics [18]. Additionally, diabetes affects microglial function; a review that examined 267 articles found that diabetes modulates microglia by affecting their secretion of a wide variety of cytokines and chemokines (NF-κB, NLRP3 inflammasome, fractalkine/CX3CR1, MAPKs, and Akt/mTOR), their metabolic reprogramming, and their increased promotion of reactive oxygen species (ROS) [19]. Brabazon et al. showed that insulin reduced the pro-inflammatory M1 microglial phenotype [20]. They exposed cultured microglia to the pro-inflammatory stimulus of lipopolysaccharide, and then, after administering insulin, they saw significantly reduced production of NO, ROS, and TNF, which showed that insulin diminished the pro-inflammatory M1 phenotype of microglia. Haas et al. added insulin to microglia in culture and showed an increase in phosphorylated Akt Ser473, which is an M2 microglial protein, thereby reflecting a switch from the pro-inflammatory M1 microglial isoform to the anti-inflammatory M2 isoform [21].

Insulin resistance is another mechanism for this association, and its role in the pathogenesis of AD cannot be underestimated. It involves the endoplasmic reticulum (ER), the unfolded protein response (UPR), autophagy, and mitochondrial function. The complex molecular biology involved in insulin resistance has a key role in the pathogenesis of AD and, therefore, in the quest for its cure. This is not the place for an exhaustive description of the details of the complex mechanisms; the following is a synopsis of the actions of the various components.

The endoplasmic reticulum (ER) is a major player because it regulates proteostasis, lipid metabolism, gluconeogenesis, and calcium signaling; moreover, it is the site where the early steps of insulin biosynthesis occur [22]. Disturbances in ER homeostasis are referred to as “ER stress” [23]. Hyperglycemia, hypoxia, and ROS lead to ER stress and the UPR may follow [24]. Three proteins, protein kinase-like ER kinase (PERK), inositol-requiring 1 (IRE1), and activating transcription factor 6 (ATF6), function as sensors controlling the UPR during ER stress [25]. Upon their activation, these three sensor molecules trigger a signaling cascade leading to the UPR, from which downstream effectors promote an adaptive response, feedback control, and regulation of cell fate. The adaptation initially involves activation of molecular chaperones and folding enzymes in order to enhance protein folding. This leads to reduced ER workload through mRNA degradation and attenuation of translation, as well as elevated ER-associated protein degradation and the clearance of unwanted proteins through autophagy. If the UPR becomes hyperactivated, e.g., from hyperglycemia or ROS, the UPR regulators switch off so as to resolve the unwanted effects.

As occurs with so many intercellular and intracellular functions, there are multiple interconnectivities between the ER, the UPR, autophagy, mitochondrial function, and insulin resistance. Another protein, the mitogen-activated protein kinase (MAPK), orchestrates diverse events related to AD, such as tau phosphorylation, ROS, neurotoxicity, neuroinflammation, and synaptic dysfunction, and there is cross-talk between the MAPK signaling pathways and the UPR; moreover, mitochondria, which control so many aspects of cellular metabolism, are physically connected with the ER through specialized proteins in a region that is called the mitochondria-associated ER membrane (MAM). One of these proteins is mitofusin 2 and links the ER and mitochondria through insulin signaling [26].

In brief, the UPR is important in the pathogenesis of AD in two ways as follows: first, it affects both the survival of critical cells, i.e., neurons, astrocytes, and microglia, as well as cell death, i.e., neurocytotoxicity; and second, amyloid derives from unfolded proteins.

Regarding the actions of insulin in the brain, IRE 1 has a crucial function in insulin biosynthesis, is activated in response to ER stress, and helps maintain protein folding; IRS is central to insulin signaling and glucose regulation. 

Regarding lithium, one of its benefits is that of the reversal of insulin resistance, which is a fundamental feature of AD [27]. Lithium restored insulin sensitivity in diabetic rats [28], and lithium increased, by approximately 2.5-fold, the transport of glucose induced by insulin [29]. Inhibitors of GSK3β, such as lithium, also produced improvements in whole-body insulin sensitivity in insulin-resistant animals [30]. Nevertheless, in this respect lithium is a two-edged sword because it inhibits IRE1, which is required for insulin‘s biosynthesis [31]. Maurer et al. spectrophotometrically determined the activities of respiratory chain complexes I + III, cytochrome c oxidoreductase, complexes II + III, succinate dehydrogenase, and complex IV [cytochrome c oxidase (COX)], as well as those of the mitochondrial matrix enzyme citrate synthase in postmortem human brain cortex homogenates following exposure to lithium. Activities of complexes I + III and of complexes II + III were dose-dependently increased by lithium with maximum values at 1 mM (165%, *p* = 0.03, and 146%, *p* = 0.00002, for controls). Activity of succinate dehydrogenase was raised at higher drug concentrations (maximum 220%, *p* = 0.01, versus controls). In contrast, activity of COX was not significantly affected by lithium [32]. Valproate also participates with lithium. Bachmann et al. found that long-term treatment with both lithium and valproate enhanced the cell respiration rate, mitochondrial membrane potential, and mitochondrial oxidation in cell culture; in vivo studies showed that both lithium and valproate protected against the methamphetamine-induced reduction in mitochondrial cytochrome *c*, the ratio of mitochondrial Bcl-2:Bax, and mitochondrial cytochrome oxidase (COX) activity [33]. Zhang et al. provided further information about the benefit of valproate, which down-regulated the expression of multiple histone deacetylators (HDACs) both in vivo in AD mouse models and their cultured cells; valproate also decreased the expression of APP secretases through the JNK pathway, reduced Aβ deposition in both the AD cell and mouse models, and significantly improved cognitive function in AD mice [34].

In summary, aggressively treating diabetes, if present, should contribute to the reversal of dementia; moreover, even if there is no diabetes, insulin resistance can be overcome through the use of intranasal insulin.

## 4. Vascular Pathology and AD

Many studies have shown vasculopathy to be associated with the progression of MCI, and diabetes is merely one among a long list of risk factors for vascular disease. Among 298 patients with MCI who were followed for five years, percentages were significantly higher at baseline in those with progression to AD than in those without progression as follows: for diabetes, +9.7%; for hypercholesterolemia, +5.9%; for cardiovascular disease, +9.9%; and for hypertension, 10.5% [15]. A literature review that included five reports of patients with both MCI and cerebrovascular disease showed a RR of 1.61 for that association [6]. The same review included three reports of patients with both atrial fibrillation and MCI that progressed to dementia; for that association, the RR was 2.60. Vascular risk factors (VRFs) were considered to have been treated if they had received specific medication at baseline. Compared with the subjects with untreated VRFs, subjects with some or all VRFs that were treated had lower risks of incident AD; moreover, subjects with all VRFs treated had less AD than subjects with only some VRFs treated. Among 406 patients with MCI, there were 106 who developed AD over a one-year period [35]. In that study, conversion to dementia was significantly associated with atherosclerotic plaque severity and intimal thickness. The OR for progression from MCI to dementia was higher by 4.64 times in subjects with coronary artery disease and 4.31 times in persons with a history of cerebrovascular disease [36]. Similar data were reported by others [6,37]. Associations of neuropathology with both atherosclerosis and arteriolosclerosis were reported for 1143 subjects [38]. The OR was 1·33 between atherosclerosis, i.e., macrovascular disease, and AD; moreover, atherosclerosis was associated with significantly worse scores in cognitive domains for episodic memory, semantic memory, perceptual speed, and visuospatial abilities. Arteriolosclerosis, i.e., microvascular disease, was also associated with worse scores for global cognition, episodic memory, semantic memory, working memory, and perceptual speed. 

In brief, the data show that treating vascular pathology, if present, should contribute to the reversal of dementia.

## 5. Dyslipidemia and AD

Although the brain has ~25% of the total amount of the body’s cholesterol, little diet-derived cholesterol enters the brain, and all cholesterol accumulated in the brain during the period of rapid myelination can be explained by its local synthesis by astrocytes [39]. Nevertheless, patients with increased blood cholesterol and MCI that progressed to dementia had a RR of 1.61 for that association [6]. An epidemiological study found that hypercholesterolemia in MCI patients was associated with transition from MCI to AD; treatment that lowered cholesterol levels reduced the risk of MCI transitioning to AD. Analysis of 21 studies showed a reduced rate of Alzheimer’s dementia associated with statin use, with an OR of 0.68 and without any differences when analysis was stratified by sex or by the use of lipophilic and hydrophilic statins; there was a borderline difference (*p* = 0.05) for use of high-potency statins (20% reduction in dementia) as compared with low-potency statins (16% reduction) [40]. An earlier meta-analysis had produced similar results from 184,666 incident cases, showing that the use of statins was associated with a reduced risk of Alzheimer’s dementia (RR = 0.81), and for every one year of use, there was a 20% decreased risk of dementia [34].

In brief, the data show that treating dyslipidemia, if present, should contribute to the reversal of dementia.

## 6. Hypertension in MCI Patients Who Did Not Revert to Having Normal Cognition

There is mixed evidence regarding whether patients with MCI and hypertension have more conversion to dementia than normotensives. Thus, one review showed a HR of 1.39 for the association with hypertension in 1516 cases of dementia, and another one that reviewed six reports gave a pooled HR of 1.21 [6]; however, Cooper et al., in a systematic review of 76 reports, found consistent evidence in studies of people with any-type MCI that hypertension did not predict all-cause dementia [15]. However, Cooper et al. did note that the only large, higher quality epidemiological study to investigate conversion from amnestic MCI to Alzheimer’s dementia found that hypertension was a significant predictor of subsequent dementia. Ravaglia et al., reporting on 165 patients followed for three years, found that each 10 mm decrement in either systolic or diastolic pressure was significantly predictive of a decreased risk of MCI conversion to dementia, suggesting that the treatment of hypertension in patients with MCI might decrease the incidence of dementia.

However, the essential query is not whether hypertension in patients with MCI predicts the conversion to dementia, which it does, or whether dementia cases have more hypertension, but it is whether antihypertensives ameliorate established AD. Several reports show benefits for dementia from treatment of hypertension, but several do not. The explanation for the conflicting evidence is probably that of the varied effects of different classes of antihypertensive drugs. In AD model mice, angiotensin receptor blockers (ARBs) such as losartan, valsartan, and telmisartan prevented cognitive decline; however, AD model mice are imperfect analogs of human AD. Data from humans are more persuasive. The electronic health records of 14,269 patients with MCI included 1247 (8.7%) who progressed to AD and 2501 (17.5%) who had any form of dementia [41]. β-blocking drugs, either alone or in combination with diuretics, caused a significant decrease in dementia, but a benefit for dementia from other antihypertensive drugs classes was not significant. Shah et al., reviewed 12 studies, most involving patients with AD or vascular dementia; only ACE inhibitors and diuretics significantly reduced the risk of progression of dementia in the majority of those studies [42].

Antihypertensive drugs provide benefits beyond that of decreased dementia, e.g., diminished amyloid deposition. This was examined through the high-throughput screening of 55 commercially available antihypertensive drugs, which identified four compounds that significantly reduced Aβ_1-42_ oligomerization in a dose-dependent manner [43]. However, the four compounds of furosemide (diuretic), nitrendipine (calcium channel blocker), candesartan cilextil (ARB), and diazoxide (vasodilator) showed no detectable Aβ-lowering activities in primary neuron cultures. Nevertheless, furosemide, nitrendipine valsartan and candesartan cilextil prevented the oligomerization of both Aβ_1-40_ and Aβ_1-42_ in vitro; moreover, furosemide also dissociated pre-aggregated Aβ_1-42_ oligomers. Afflek et al. examined human brain tissue from cases medicated for hypertension, involving 46 AD cases and 33 controls matched for cerebrovascular disease [44]. Multivariate analyses showed that antihypertensive medication use was associated with a less extensive spread of AD proteins throughout the brain.

Interestingly, there is also increased dementia in subjects with prehypertension (≥120/80–<140/90); it was lower in black persons (HR 1.17) than in white persons (HR 1.35) [45].

In brief, the data show that treating hypertension, if present, should contribute to the reversal of dementia.

## 7. Inflammation and AD

The level of C-reactive protein (CRP), which can reflect either cerebral or systemic inflammation or both of these, was examined in a cohort of 12,336 participants with a baseline age of 56.8 years. For each standard deviation (SD) increase in CRP, there was a decline over 20 years of −0.035 SD on the cognitive composite score; participants with a midlife CRP in the top quartile had a 7.8% steeper cognitive decline compared to that of participants in the lowest quartile, and elevated CRP in midlife was consistently associated with declines in memory [46]. Immune activation has a role in both the pathogenesis of AD and its progression; it may be a primary event in the brain for the development of AD but may also be mediated by systemic inflammation [47]. Genome-wide association studies (GWASs) showed that genes encoding immune receptors link with AD [47]. One of the ways whereby cerebral inflammation contributes to causing AD is because inflammatory mediators up-regulate beta secretase that promotes APP processing and the release of Aβ [48].

Regarding inflammation and nonsteroidal anti-inflammatory drugs (NSAIDs), it is of interest to mention that 15 epidemiological studies showed the prevalence of Alzheimer’s disease (AD) as being reduced in patients with rheumatoid arthritis (RA) who had been treated with NSAIDs [49]. One of those studies contained 691 AD subjects who were compared with 973 family members and showed less NSAID use among AD persons with an odds ratio (OR) of 0.64; moreover, for APOEɛ4 carriers versus non-carriers, the OR = 0.49 [50]. Another study, with 104 AD subjects, showed the hazard ratio (HR) = 0.42 for those with ≥2 years’ exposure to NSAIDs [51]. A third study had 74 AD subjects and found a risk ratio (RR) of 0.74 for those who had had exposure to NSAIDs for two or more years [52].

The gut microbiome is now recognized as playing a critical role in neurodegeneration and AD progression, doing so by affecting Aβ oligomers and plaques, tau aggregates, and neuroinflammation; manipulation of the gut microbiome with antibiotics has resulted in both a reduced progression of AD and a reduction in Aβ deposition [53]. The complex mechanisms that might modulate the connection between the gut microbiome and AD were summarized by Kohler et al., in whose model there is increased translocation due to a leaky gut (either caused by aging or environmental factors) of Gram-negative bacteria, lipopolysaccharide fragments that lead to the production of ROS, toxic catabolites from bacteria, increased levels of quinolinic acid, changes in energy metabolism, and insulin resistance, and there are reduced levels of short chain fatty acids, so the astrocyte–neuron glutamate–glutamine shuttle is impaired [54]. These mechanisms, all related to the gut microbiome and intestinal inflammation, would act together to reduce the clearance of Aβ from the CNS and to increase microglial activation that promotes neurotoxicity.

In brief, the data show that treating systemic inflammation, if present as shown by increased CRP, should contribute to the reversal of dementia.

## 8. Underweight in MCI Patients Who Did Not Revert to Having Normal Cognition

A number of reports show that body weight differs between those patients who progress to AD in contrast to those who do not progress. For patients with cognitive impairment, being underweight was associated with progression to AD [55]. A literature review that involved patients in whom MCI had progressed to dementia and who had had their body mass index (BMI) assessed showed a RR of 0.85 for that association [6]. That was confirmed by a report of almost two million people showing that being underweight in both middle age and old age carried an increased risk of dementia over two decades and by another study showing that over a one-year period, a lower baseline BMI was associated with significant declines in cognitive performance [56]. Furthermore, in 521 patients with mild-to-moderate AD, rapid cognitive decline was associated with malnutrition [57]. That was also seen in a study of 414 patients with probable AD, for whom weight loss was defined as a loss of 4% or more during the first year of follow-up, and rapid cognitive decline was the loss of 3 points or more in MMSE over 6 months [58]. A total of 87 (21.0%) of 414 lost 4% or more of their initial weight during the first year; rapid decline of weight affected 57.6% of the patients after a median follow-up of 15.1 months, and after controlling for potential confounders, it was a significant predictor factor of rapid weight loss when the HR = 1.50.

Related to the association of possible malnutrition and the progression of MCI to dementia was vitamin D deficiency (OR 3.13), which was present in 14% of 250 patients with MCI in Thailand; it is also noteworthy that in AD model mice, a diet enriched with vitamin D was associated with decreased levels of amyloid plaques in their brains [59]. Folate deficiency [15,60,61,62] may also be associated with malnutrition; the OR was 0.38 for lower folate being present at baseline and 0.44 for lower folate being present at the five-year follow-up. Higher folate levels at baseline in females predicted a lower conversion rate to dementia; and users of folate had lower grades of periventricular hyperintensities and lower grades of deep white matter lesions as compared to non-users [61].

The effects of a Mediterranean diet were examined in two systematic reviews; both found that the diet decreased conversion from MCI to AD [15]. Benefit from dietary modification might be mediated by saponins, which provide neuroprotective mechanisms, including those of free radical scavenging, the modulation of neuroprotective signaling pathways, the activation of neurotrophic factors, the modulation of neurotransmitters, the inhibition of BACE1 enzyme, and tau hyperphosphorylation [63].

Wu et al. found that short-chain fatty acids (SCFAs), which have fewer than six carbons and are largely derived from the metabolism of dietary fiber by gut microbes, had strong predictive ability for the conversion from amnestic MCI to AD [64]. SCFAs include acetic acid, propionic acid, butyric acid, and valeric acid; they cross the BBB, and they promote inflammation in the brain by enhancing the function of microglia; they also provide energy to cells because they enter the citric acid cycle in the mitochondria to generate ATP. Both butyrate and propionate are active in neurons [65]. Sodium butyrate is an inhibitor of histone deacetylase, so it enhances histone acetylation that promotes gene transcription by means of which it significantly facilitates associative memory function when administered to AD model mice [65,66]. More recently, a study reported that the modified Mediterranean-ketogenic diet modulated the intestinal microbiome, and the resulting increase in SCFAs in MCI patients was associated with the improved AD biomarkers in CSF [67]. Thus, a decreased formation of SCFAs in the intestine might be crucial mediators between the intestinal microbiome and AD [64].

In brief, the data show that treating reduced weight and correcting deficiencies of folate and vitamin D, if those are present, and following a Mediterranean diet should contribute to the reversal of dementia.

## 9. Mitochondrial Abnormalities and AD

Impaired mitochondrial function affects virtually every aspect of brain function, and it has been known for several decades that there is mitochondrial dysfunction in AD, which includes the loss of mitochondrial structural and functional integrity, impaired mitochondrial biogenesis and dynamics, altered mitochondria interaction with the endoplasmic reticulum, altered mitochondrial proteostasis, and increased mitophagy [68]. Parenthetically, it is worth noting that all of those mitochondrial dysfunctions are attenuated by lithium. It has been noted above that valproate partners with lithium in this effect upon mitochondrial function. In AD, mitochondria had reduced numbers, were morphologically abnormal, had fewer genes encoding subunits of the electron transport chain, and had decreased activities of the TCA cycle; moreover, the PPARɤ coactivator 1-α that regulates mitochondrial biogenesis also had reduced levels [69].

Reactive oxygen species (ROS) and nitrogen species are formed in mitochondria; therefore, mitochondrial action may mediate neurotoxicity and thus can be a central element in the pathogenesis of AD.

Detection of acquired mitochondrial abnormalities may be problematic short of a tissue biopsy with observation by electron microscopy of the organelle; however, clues might come from plasma enzyme deficiencies, including those of cytochrome oxidase, pyruvate dehydrogenase, α-ketoglutarate dehydrogenase, and ATP synthase (shown by the reduced level of ATP).

In brief, if mitochondrial abnormalities can be established, then improving mitochondrial function with lithium should contribute to the reversal of dementia.

## 10. Transforming Growth Factor β (TGF-β)

Although there is an increase in TGF-β1 with advancing old age, there is a concomitant decrease in its receptor, TGFR2, in the brain, resulting in the reduced neurotrophic efficacy of TGF-β. In light of that, it is perhaps paradoxical that the level of TGF-β is not reduced in MCI [70], but the level of FAMC in AD, a key molecule in the formation of TGF-β, is reduced by ~30–50% [70,71]. Therefore, if in a particular patient with AD the plasma level of TGF-β is reduced, then its levels should be raised [72], which may be accomplished by the administration of fluoxetine, which approximately doubles the levels [73]. Parenthetically, blood levels of TGF-β can be obtained in commercial laboratories.

In brief, if TGF-β levels are decreased in patients with AD, then raising them by the administration of fluoxetine should contribute to the reversal of dementia.

## 11. Wnt/β-Catenin and AD

Studies by Inestrosa et al. showed that Wnt/β-catenin is involved in regulating synaptic plasticity and maintaining BBB integrity; Inestrosa et al. also found that activated WNT/β-catenin signaling prevented neural toxicity caused by Aβ, that WNT/β-catenin participates in a normal degree of tau phosphorylation and in learning and memory, and that WNT/β-catenin dysfunction results in Aβ production and aggregation [74]. Tay et al. followed 14 subjects with MCI and 74 with mild-to-moderate AD and measured the scores for the clinical dementia sum of boxes (CDR-SB) at baseline and after one year and assessed the correlations between changes in the CDR-SB and serum levels of Dickkopf-1 (Dkk-1), which is an antagonist of Wnt [75]. Decreased levels of Wnt as shown by the increase in Dkk-1, which is an antagonist of Wnt/β-catenin, were significantly associated with progressively higher CDR-SB scores (indicating more impairment) among patients with AD but not among patients with MCI. Also important, a reduction in canonical Wnt signaling promoted tau hyperphosphorylation, which may be causal of AD [76].

Three reports showed that doxycycline, a commonly used antibiotic, raised levels of Wnt/β-catenin. Noting that Wnt signaling is established as an essential bone-promoting mechanism associated with bone healing, Song et al. showed that both Wnt7b and doxycycline increased the density of the callus at the site of a fracture [77]. Zhang et al. also found that doxycycline increased bone formation, and this was accompanied by up-regulation of β-catenin and TGF-β [78]. Gomes et al. reported that doxycycline decreased the immunostaining of Dikkopf-1 (Dkk-1), reflecting an increase in Wnt, by as much as 63% and increased the immunostaining of Wnt-10b by as much as 150% [79]. Parenthetically, blood levels of canonical Wnt can be obtained in commercial laboratories.

In brief, the data show that raising levels of Wnt/β-catenin, if low, should contribute to the reversal of dementia.

## 12. EMT

EMT refers to the transformation of cells from the epithelial to the mesenchymal condition. In AD, there is a down-regulation of neurons with the M phenotype. This was shown by Liu et al., who immuno-stained FAM3C, a key molecule in causing the E-to-m transition, and found it to be 45% lower in AD brains than in controls [80]. Those findings were confirmed in studies by Watanabe et al., who also saw an overall 46% reduction in FAM3C; it was reduced by 27% in Braak stages 3–4 compared with non-demented controls having Braak stages 1–2, but it was reduced by 51% in Braak stages 5–6 [81]. Hasegawa et al. demonstrated that TGF-β induced the neuronal expression of FAM3C [82].

In brief, treatment with fluoxetine, as described above, that increases the levels of TGF-β may lead to an increase in FAM3C, then in M-neurons, and finally to neurogenesis, which should benefit the attempt to the reverse AD. 

## 13. Metabolic Syndrome and AD

Metabolic syndrome is characterized by the concurrence of three or more of the following conditions: diabetes, dyslipidemia with either low HDL cholesterol or high triglycerides, hypertension, and increased waist circumference. Unsurprisingly, since diabetes, hypertension, and dyslipidemia have all been shown to be related to the progression of MCI to dementia, several reports have indicated that metabolic syndrome is a risk factor for the progression of MCI to dementia. For example, in the Italian Longitudinal Study on Aging, the HR was 4.4 comparing those subjects with MCI who progressed to having dementia versus with those without that progression [83]. In another example, after excluding individuals with known AD, Vanhanen et al. randomly chose 998 elderly subjects, aged 69–78; 418 of them had metabolic syndrome, and 45 (4.7%) were found to have either probable or possible AD [84]. For those 45 individuals, multivariate logistic regression analysis including apolipoprotein E4 phenotype, education, age, and total cholesterol, metabolic syndrome was significantly associated with AD (OR 2.46); moreover, even after excluding diabetic subjects, metabolic syndrome was still significantly associated with AD (OR 3.26).

Treatment should be directed, in particular, to hypertriglyceridemia, increased waist circumference, and hypertension, which were the components that gave the highest rate of progression to dementia [83].

In brief, the data show that treating the components of the metabolic syndrome, if present, should contribute to the reversal of dementia.

## 14. Disturbed Circadian Rhythm in AD: Causal Factor or Epiphenomenon? If a Causal Factor, Then Treatment May Benefit Patients with AD

An abnormal circadian rhythm producing an impaired sleep/wake cycle importantly contributes to causing AD. Weldemichael and Grossberg noted that in AD, nocturnal sleep disturbance is associated with the degree of dementia [85]. Homolak et al. pointed to the fact that some of the key processes involved in the pathogenesis of AD show the involvement of the circadian rhythm [86]. However, including it as a causal factor that should be addressed in individual patients with AD raises the question of whether this abnormal circadian rhythm is a cause or an epiphenomenon of AD. That question has no definitive answer; however, an abnormal circadian rhythm is included here as a possible causal factor because several studies show that it is predictive of future MCI and, in one report, of dementia.

Sleep–wake cycles, i.e., circadian rhythms, may be assessed by an actigraph, which is an instrument worn on the wrist, to monitor movement over an extended period of time. This was used to collect data from 1282 healthy women, with a mean age of 83 years, each of whom completed a neuropsychological test battery and 4.9 years later had her clinical cognitive status adjudicated by an expert panel; it found that 195 (15%) women had developed dementia and 302 (24%) had developed MCI [87,88]. All analyses of the data were adjusted for demographics, BMI, functional status, depression, medications, alcohol, caffeine, smoking, health status, and co-morbidities, and they showed that when peak levels of subjects’ movements occurred later in the day than average, i.e., had a delayed circadian activity, there was an increased risk of MCI or dementia (OR 1.83); moreover, when there was a decreased amplitude of circadian rhythm (the difference between the peak and trough levels of activity), the odds for dementia or MCI also increased (OR 1.57). A later analysis of the same data confirmed the higher risk of dementia but not of MCI [89]. Analysis of data from 763 participants who wore an actigraph in the Women’s Health Initiative also determined the relation between rest–activity measures and centrally adjudicated MCI or dementia [90]. Reduced overall rhythmicity, lower amplitude and activity levels, and activity later in the day were all associated with higher risk of MCI and probable dementia; moreover, women with a lower amplitude of the circadian rhythm also exhibited faster cognitive decline over follow-ups.

In cognitively normal participants, with a mean age of 62.9 years, with a parental history of sporadic AD and who were thus at risk of future AD, worse subjective sleep quality and daytime somnolence were associated in CSF with lower Aβ_42_, Aβ_40_, and higher total tau:Aβ_42_ and p-tau:Aβ_42_ [91]. Others found that the progression of AD was mirrored by sleep/wake variables [92]. The mechanism linking decreased sleeping time and AD is that of the reduced removal of potential toxins, particularly Aβ, in the awake brain in which there is reduced volume of the interstitial space and, therefore, a higher concentration of toxins. The cortical interstitial space was 66% higher in sleeping mice than in awake mice [93]. Another study showed hippocampal Aβ levels during sleep deprivation rising by 33.7% and, with sleep, immediately falling [94]. That diurnal fluctuation of Aβ in the brain’s interstitial fluid was also seen in humans. One night of unrestricted sleep led to a 6% decrease in Aβ_42_ levels, whereas sleep deprivation counteracted that; however, in sleep-deprived, cognitively normal subjects, the mean levels of overnight CSF Aβ_38_, Aβ_40_, and Aβ_42_ increased above baseline levels by 30% [95]. Similarly with the tau protein, in healthy men, the plasma total tau increased by 17.5% in response to sleep loss, as compared with only 1.8% during normal sleep [96].

In view of the above explanation, it is surprising that therapy with light is also beneficial. Photobiomodulation (PBM) is the mechanism by which nonionizing optical radiation in the visible and near-infrared spectral range is absorbed by endogenous chromophores to elicit photophysical and photochemical events; moreover, PBM therapy (PBMT) is a photon therapy that uses nonionizing forms of light to cause physiological changes and therapeutic benefits [97]. Among 18 randomized controlled trials with persons with dementia, light therapy applied during awake hours reduced nighttime awakenings and enhanced sleep quality [40]. An infrared laser (780–1100 nm) triggered increased cerebral blood flow, mitochondrial activation that enhanced neuroprotection, and the activated N-methyl-D-aspartate receptor (NMDAR) that decreased the intracellular overload of Ca^2+^ and prevented excitatory neurotoxicity, and it reduced excitatory neurotransmission in the hippocampus [98]. In response to light at 808 nM, microglia exposed to Aβ switched from glycolysis to enhanced anti-inflammatory activity, and when microglia were co-cultured with neurons and exposed first to Aβ and then to light, ROS production was decreased with less neurotoxicity [99]. Light induces beneficial pathways including those of extracellular signal-regulated kinase (ERK), mitogen-activated protein kinase (MAPK), and protein kinase B (Akt), and it activates neurotrophic factors and secretases [100,101]. Exposing the frontal skull of rats to a light-emitting diode led to improvements in spatial memory as well as in behavioral and motor skills after inoculation of the hippocampus with Aβ that had decreased them.

Thus, although there is benefit from apparently opposing therapeutic approaches, this is because each approach induces different mechanisms.

## 15. Discussion

This article has considered the biological changes that (1) accompany the transition from MCI to AD, (2) are present in AD but not in controls, and (3) are not present in MCI patients who revert to having normal cognition, yet they remain in those whose MCI persists, which comprise a group that forms the pool of patients among which are those who will later develop dementia. The clinical significance is that if those biological changes are present in an individual who has Alzheimer’s dementia, then correcting a sufficient number of those factors may contribute to curing the dementia. The causal factors considered in this article are only those that are modifiable by a therapeutic intervention, and they are cerebrovascular impairment, circadian rhythm abnormalities, depression, diabetes mellitus, hyperlipidemia, hypertension, being underweight, malnutrition including deficiencies of folate and vitamin D, metabolic syndrome, mitochondrial abnormalities, TGF-β deficiency, and WNT/β-catenin deficiency. Their presence should be sought using standard methods and their correction may be achieved by standard treatments, although some therapeutic approaches are mentioned in this article.

It might be considered that if it has already been shown that treating a particular causal factor did not benefit AD, then that treatment has no role in curing the dementia. However, this article holds otherwise because the essential question remains of how many reversible causes in any particular patient with dementia need to be treated in order to achieve a cure? Does that number of causes require treating one, two, three, or more than three? Data suggest that it is more than one, which is why a previously negative report about any one patient applied singly does not deny it a role in combination treatment. Single treatments with either lecanemab or aducanumab did not cure the dementia despite there being many promising preclinical data for their potential efficacy, and many other compounds with equally promising preclinical data were also shown in clinical trials to be ineffective. Thus, background data indicate that curative therapy for AD requires the administration of more than one drug. For a clue as to how many drugs are required, one must look at conditions other than that of AD. A study on diabetes provided useful data [102]. That randomized, controlled trial examined the risk of future cardiovascular disease (CVD) in diabetic participants who had no history of CVD and contained three groups as follows: those with a low risk because of having only 0–1 risk factors, those with an intermediate risk because of having 2–3 risk factors, and those with a high risk because of having 4 risk factors. Intervention was carried out for four years, during which time each group had significantly fewer major cardiac events and deaths than expected; as the number of risk factors being addressed increased, the CVD prognosis progressively improved. In brief, the response to the question “how many reversible causes that are found in any particular patient with dementia, need to be treated in order to achieve a cure?” seems to be that the data suggest that the correction of ≥3 of the factors listed in this present article (see Table 2) might provide a strong likelihood of curing the dementia.

Adherence to treatment is obviously relevant and was studied in patients enrolled in a Medicare Advantage Prescription Drug plan regarding how outcomes were affected by ≤80% adherence to medications prescribed for treatment of diabetes, hypertension, and hyperlipidemia in a study population of 99,774 individuals with a mean age of 71.0 years [103]. Compared with patients who missed zero adherence measures, those who missed one measure had 23%–33% increased odds of cognitive decline (for any decline, OR = 1.23; for dementia, OR = 1.33; for Alzheimer’s disease, OR = 1.27; all *p* values < 0.01); patients who missed 2–3 measures had 37–96% increased odds of cognitive decline; and patients who missed ≥4 adherence measures had the greatest odds of cognitive decline (for any decline, OR = 1.64; for dementia, OR = 2.05; for Alzheimer’s disease, OR = 2.48; all *p* values < 0.01). The potential likelihood of curing AD using the approach advocated in this article requires adherence to the administered medications, which must be accounted for in a clinical trial that is intended to evaluate the validity of the approach.

## 16. Conclusions and Summary

The cure of Alzheimer’s dementia may be accomplished by treating ≥3 of the reversible factors that are present in individual patients. Those factors are identified as being present in a higher number of MCI subjects who did not revert to having normal cognition than in those who did revert. As such, this represents personalized medicine.

The reversible factors include circadian rhythm abnormalities; diabetes; hyperlipidemias; hypertension; inflammation; cerebral vasculopathy; being underweight; low levels of vitamin D, folate, and niacin; reduced TGF-β; reduced Wnt/β-catenin; and metabolic syndrome. Addressing these issues with standard therapies in patients with AD for whom ≥3 of these factors are identified should achieve a high likelihood of curing the dementia.

A clinical trial is necessary to validate both the accuracy and safety of the suggested approach.

## Figures and Tables

**Table 1:** Generic approach to determine conditions to treat if present in an individual patient after transition from MCI to dementia.

Condition	Present at MCI	Present at Dementia.	Treat at Dementia?
Depression	+	+	+/− **
Diabetes	+	+	+
Hypertension	−	+	+ *
Lipids ↑	−	+	+
CRP ↑	+	+	+
Weight ↓	+	+	+
Wnt ↓	−	+	+ *
Metabolic synd.	−	+	+ *
Circadian rhythm ↑	+	+	+

* although absent in MCI, it is treated in dementia since data show its association with dementia; ** see text section on Depression, regarding antidepressants aggravating risk of dementia.

**Table 2:** Elements that participate in the pathogenesis of Alzheimer’s dementia.

Causal Element	Human?/Benefit/Refs.	Clinical Trial?
Depression	Yes/+/− */[1,7]	No
Diabetes	Yes/+++/[6,12,14,15]	No
Insulin resistance	Yes/++/	No
Hyperlipidemia	Yes/++/++/[6]	No
Hypertension	Yes/+ or −/[6,14,15]	No
Inflammation	Yes/++/[55]	No
Mitochondria	Yes/++/[78,79,80,81,82,83]	No
Nutrient deficiency	Yes/++/[79,81]	No
TFG β	Yes/++/[84,85]	No
Wnt/catenin-β	Yes/++/[88,89]	No
Metabolic syndrome	Yes/+/[97,98]	No
Circadian rhythm	Yes/+++/[99,100,101,102,103]	No
Underweight	Yes/+++/[52,53,54,55,56]	No
Vascular abn’s	Yes/++/[6,13,15,38]	No

Benefit from treatment is shown as follows: − if none, + if slight, ++ if moderate, and +++ if marked. Note that these are causal elements vis-à-vis the functional capacities of neurons, neural tracts, and hippocampal function, which are the ultimate cause of Alzheimer’s dementia. * see text section on Depression, regarding antidepressants aggravating risk of dementia.
